# Declining incidence and improving survival of ocular and orbital lymphomas in the US between 1995 and 2018

**DOI:** 10.1038/s41598-024-58508-7

**Published:** 2024-04-03

**Authors:** Ahmad Samir Alfaar, Yacoub A. Yousef, Matthew W. Wilson, Omneya Hassanain, Vinodh Kakkassery, Mohanad Moustafa, Ahmad Kunbaz, Amanne Esmael, Olaf Strauß

**Affiliations:** 1 Medical Neuroscience PhD Program, Charité - Universitätsmedizin Berlin, Corporate Member of Freie Universität, Berlin Institute of Health, Humboldt-University, 10117 Berlin, Germany; 2 Department of Ophthalmology, The Royal Liverpool University Hospital, Liverpool, UK; 3 Department of Surgery/Ophthalmology, King Hussein Cancer Center, Amman, Jordan; 4 St. Jude Children’s Research Hospital, Memphis, TN USA; 5 Research Department, Children’s Cancer Hospital –Egypt, 57357, Cairo, Egypt; 6 Ophthalmology Department, University Hospital of Schleswig-Holstein, Lübeck, Germany; 7 Ophthalmology Department, Klinikum Chemnitz, Chemnitz, Germany; 8 Ophthalmology Department, University Hospital Hairmyres, East Kilbride, Scotland, UK; 9 Ophthalmology Department, Istanbul Medeniyet University, Istanbul, Turkey; 10 Ophthalmology Department, Cairo University, Cairo, Egypt; 11 Experimental Ophthalmology, Charité - Universitätsmedizin Berlin, Corporate Member of Freie Universität, Berlin Institute of Health, Humboldt-University, 10117 Berlin, Germany; 12 Department of Ophthalmology, University of Tennesse Health Science Center, Hamilton Eye Institute, Memphis, TN USA

**Keywords:** Ocular, Orbital, Lymphoma, Epidemiology, Incidence, Survival, Trends, NAACCR, Public health, Epidemiology, Cancer epidemiology

## Abstract

This epidemiological study examined ocular and orbital lymphomas in the United States from 1995 to 2018, using data from the North American Association of Central Cancer Registries database of 87,543 patients with ocular and adnexal malignancies. We identified 17,878 patients (20.4%) with ocular and orbital lymphomas, with an age-standardized incidence rate (ASIR) of 2.6 persons per million (ppm). The incidence was the highest in the orbit (ASIR = 1.24), followed by the conjunctiva (ASIR = 0.57). Non-Hodgkin B-cell lymphoma was the most prevalent subtype (85.4%), particularly marginal-zone lymphoma (45.7%). Racial disparities were noted, with Asia–Pacific Islanders showing the highest incidence (orbit, 1.3 ppm). The incidence increased significantly from 1995 to 2003 (Average Percent Change, APC = 2.1%) but declined thereafter until 2018 (APC = − 0.7%). 5-year relative survival (RS) rates varied, with the highest rate for conjunctival lymphoma (100%) and the lowest for intraocular lymphoma (70.6%). Survival rates have generally improved, with an annual increase in the 5-year RS of 0.45%. This study highlights the changing epidemiological landscape, pointing to initial increases and subsequent decreases in incidence until 2003, with survival improvements likely due to advancements in treatment. These findings underscore the need for further research to investigate the root causes of these shifts and the declining incidence of ocular lymphoma.

## Introduction

The orbit encompasses diverse anatomical structures with distinct histological origins, giving rise to a wide range of tumors that exhibit variations based on their specific origins. The eye and its adnexal lymphomas are caused by the proliferation of lymphoid tissues within the ocular region. Among these lymphomas, ocular adnexal lymphoma is the most prevalent subtype, constituting more than 24% of all malignancies originating in the orbit, particularly among the elderly^1,2^. Most orbital lymphomas are of B cell origin (97%), with extranodal marginal zone B cell lymphoma representing the most frequently reported subtype, followed by diffuse large B-cell lymphoma^3^.

The incidence and epidemiological trends of ocular and orbital lymphomas are the subjects of ongoing research, with notable variations observed across different demographic groups. Historically, much of the data has been sourced from specific tertiary referral centers, often focusing on particular age groups or histological subtypes, leading to a piecemeal understanding of these conditions’ overall trends and survival outcomes^1,4,5^.

Population-based studies that provide comprehensive data on these relatively rare lymphomas are scarce. Our understanding primarily stems from retrospective analyses with limited sample sizes, which may not fully capture the incidence rates in diverse populations, including minorities and small ethnic groups. This suggests that the current incidence figures may underrepresent these demographics, underscoring the need for further research. A limited number of population-based studies have examined ocular and orbital tumours trends and incidence rates, aiming to provide accurate data on these relatively rare conditions^6–8^.

Recent studies have reported an increased incidence of ocular and adnexal lymphomas in countries such as the USA, Canada, Denmark, and South Korea^9,10^. It is crucial to update our understanding of these trends, particularly in light of recent advancements in diagnostic and treatment methodologies, particularly for diseases that could act as risk factors for lymphoma development. By investigating the incidence rates of various tumors and monitoring their recent trends, healthcare professionals can gain valuable insights into the frequency, distribution, and specific populations that should be considered for screening, thereby minimizing the risk of misdiagnosis.

Our study aimed to provide an updated, comprehensive overview of the incidence rates and trends of ocular and orbital lymphomas across different age groups and ethnicities in the United States from 1995 to 2018. We also sought to analyze the changes in the incidence and survival patterns over this period, contributing to a better understanding of the dynamics of these malignancies.

## Results

### Population characteristics and incidence

**Figure 1:**
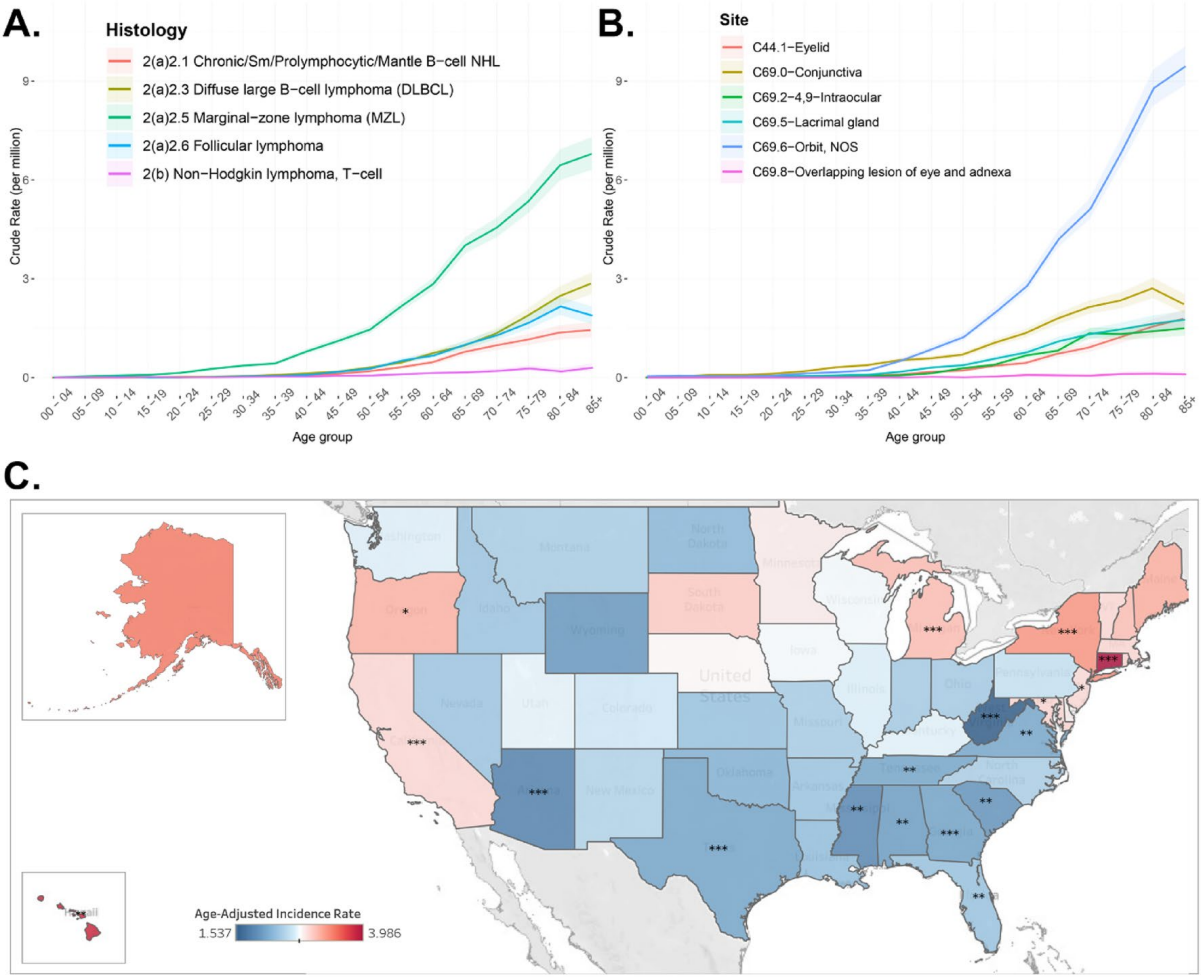
(**A**) Crude incidence rate per age group in the major histological groups. (**B**) Crude incidence rate per age group of ocular sites. (**C**) Age-standardized incidence rates in different U.S. states. *Represents the significance of state rate-ratio to US national age-standardized incidence; *p > 0.05–0,0051, **0.005–0.0005, ***p < 0.0005 .

Among 87,543 patients with ocular and adnexal malignancies retrieved from NAACCR in the study period, our study detected 17,878 (20.4%) patients affected with ocular and orbital lymphoma, representing a cumulative age-adjusted incidence rate of 2.6 (95% CI 2.59–2.67) persons per million (ppm). The incidence was highest in the orbit (1.24 ppm, N = 7445), followed by the conjunctiva (0.57 ppm, N = 8446). The incidence in males was 2.58 compared to 2.63 ppm in females, where the number of cases in females was higher than that in males (10,011 vs. 7867) (Tables 1 and 2). Patients aged between 60 and 79 years were the most affected group (N = 8598, 48.1%)(Fig. 1).

**Table 1:** Patients’ distribution and demographics.

	C441 eyelid		C690 conjunctiva		C692 4–9 intraocular		C695 Lacrimal gland		C696 Orbit, NOS		C698 Overlapping lesions		Total	
Totals with row %	1507	8.4%	3986	22.3%	1675	9.4%	2133	11.9%	8446	47.2%	131	0.7%	17,878	100%
Age groups		Col %		Col %		Col %		Col %		Col %		Col %		Col %
00–19	12	0.8%	84	2.1%	14	0.8%	10	0.5%	79	0.9%	1	0.8%	200	1.1%
20–39	62	4.1%	444	11.1%	58	3.5%	104	4.9%	256	3.0%	5	3.8%	929	5.2%
40–59	343	22.8%	1192	29.9%	356	21.3%	595	27.9%	1895	22.4%	28	21.4%	4409	24.7%
60–79	713	47.3%	1707	42.8%	918	54.8%	1039	48.7%	4149	49.1%	72	55.0%	8598	48.1%
80 +	377	25.0%	559	14.0%	329	19.6%	385	18.0%	2067	24.5%	25	19.1%	3742	20.9%
Sex														
Male	643	42.7%	1862	46.7%	717	42.8%	744	34.9%	3845	45.5%	56	42.7%	7867	44.0%
Female	864	57.3%	2124	53.3%	958	57.2%	1389	65.1%	4601	54.5%	75	57.3%	10,011	56.0%
Laterality														
Unilateral	1429	94.8%	3501	87.8%	1361	81.3%	1936	90.8%	7873	93.2%	116	88.5%	16,216	90.7%
Bilateral	41	2.7%	423	10.6%	252	15.0%	160	7.5%	475	5.6%	14	10.7%	1365	7.6%
Unknown or miscoded	37	2.50%	62	1.60%	62	3.70%	37	1.70%	98	1.20%	1	0.80%	297	1.70%
Race														
White	1292	85.7%	3248	81.5%	1485	88.7%	1812	85.0%	7336	86.9%	106	80.9%	15,279	85.5%
Black	130	8.6%	369	9.3%	82	4.9%	189	8.9%	600	7.1%	18	13.7%	1388	7.8%
American-Indian, Alaska-Native	7	0.5%	22	0.6%	1	0.1%	12	0.6%	31	0.4%	0	0.0%	73	0.4%
Asian-or-Pacific-Islander	48	3.2%	252	6.3%	84	5.0%	93	4.4%	381	4.5%	5	3.8%	863	4.8%
Unknown	30	2.0%	95	2.4%	23	1.4%	27	1.3%	98	1.2%	2	1.5%	275	1.5%
Race (NHIA v2)														
Hispanic-all-races	92	6.1%	368	9.2%	135	8.1%	200	9.4%	659	7.8%	10	0.6%	1464	8.2%
Non-Hispanic-white	1209	80.2%	2914	73.1%	1356	81.0%	1623	76.1%	6722	79.6%	96	5.7%	13,920	77.9%
Non-Hispanic-black	126	8.4%	356	8.9%	78	4.7%	186	8.7%	577	6.8%	18	1.1%	1341	7.5%
Non-Hispanic-other	54	3.6%	271	6.8%	85	5.1%	103	4.8%	404	4.8%	5	0.3%	922	5.2%
Non-Hispanic-unknown	25	1.7%	75	1.9%	20	1.2%	21	1.0%	82	1.0%	2	0.1%	225	1.3%
Unknown-NHIA	1	0.1%	2	0.1%	1	0.1%	0	0.0%	2	0.0%	0	0.0%	6	0.0%

**Table 2:** Age-adjusted incidence rates (per million).

	C44.1 eyelid	C69.0 conjunctiva	C69.2, 4, 9 intraocular	C69.5 lacrimal gland	C69.6 orbit, NOS	C69.8 overlapping lesions
Totals	0.221 (0.21–0.233)	0.592 (0.573–0.61)	0.245 (0.233–0.257)	0.312 (0.299–0.325)	1.235 (1.209–1.262)	0.019 (0.016–0.023)
Sex						
Male	0.216 (0.199–0.233)	0.604 (0.577–0.633)	0.236 (0.219–0.255)	0.243 (0.225–0.261)	1.286 (1.245–1.328)	0.018 (0.013–0.023)
Female	0.227 (0.212–0.243)	0.588 (0.563–0.614)	0.252 (0.236–0.268)	0.371 (0.352–0.392)	1.202 (1.168–1.238)	0.02 (0.015–0.025)
Laterality						
Unilateral	0.21 (0.199–0.221)	0.519 (0.502–0.537)	0.199 (0.188–0.21)	0.283 (0.27–0.296)	1.152 (1.127–1.178)	0.017 (0.014–0.02)
Bilateral	0.006 (0.004–0.008)	0.063 (0.057–0.07)	0.037 (0.032–0.042)	0.023 (0.02–0.027)	0.069 (0.062–0.075)	0.002 (0.001–0.003)
Race						
White	0.221 (0.209–0.234)	0.57 (0.551–0.591)	0.253 (0.24–0.267)	0.311 (0.297–0.326)	1.252 (1.224–1.281)	0.018 (0.015–0.022)
Black	0.182 (0.152–0.217)	0.505 (0.454–0.561)	0.112 (0.089–0.14)	0.266 (0.228–0.308)	0.862 (0.793–0.936)	0.027 (0.016–0.043)
American-Indian/Alaska-Native	0.142 (0.05–0.303)	0.439 (0.264–0.677)	0.015 (0–0.092)	0.251 (0.12–0.448)	0.617 (0.404–0.894)	0 (0–0.067)
Asian-or-Pacific-Islander	0.176 (0.128–0.234)	0.797 (0.699–0.904)	0.29 (0.23–0.361)	0.306 (0.245–0.376)	1.332 (1.198–1.477)	0.016 (0.005–0.038)
NHIAv2 race						
Hispanic-all-races	0.157 (0.125–0.194)	0.523 (0.467–0.583)	0.235 (0.195–0.28)	0.333 (0.286–0.385)	1.117 (1.029–1.21)	0.017 (0.008–0.031)
Non-Hispanic-white	0.228 (0.215–0.241)	0.571 (0.55–0.593)	0.253 (0.24–0.267)	0.306 (0.291–0.322)	1.258 (1.228–1.289)	0.018 (0.014–0.022)
Non-Hispanic-black	0.183 (0.152–0.219)	0.511 (0.458–0.568)	0.112 (0.088–0.14)	0.273 (0.234–0.316)	0.864 (0.793–0.939)	0.028 (0.016–0.044)
Non-Hispanic-other	0.179 (0.133–0.235)	0.785 (0.691–0.887)	0.265 (0.211–0.33)	0.308 (0.25–0.375)	1.281 (1.155–1.416)	0.015 (0.005–0.034)
Lymphoma subtype/WHO 2008						
Lymphoid Neoplasm	0.212 (0.201–0.223)	0.567 (0.549–0.585)	0.231 (0.219–0.242)	0.295 (0.283–0.309)	1.182 (1.156–1.208)	0.018 (0.015–0.021)
1 Hodgkin Lymphoma	0 (0–0.001)	0 (0–0.001)	0 (0–0.001)	0 (0–0.001)	0.001 (0–0.002)	0 (0–0.001)
2 Non-Hodgkin lymphoma	0.199 (0.188–0.21)	0.536 (0.519–0.554)	0.189 (0.178–0.199)	0.282 (0.269–0.295)	1.11 (1.085–1.135)	0.017 (0.014–0.02)
2(a) Non-Hodgkin lymphoma, B-cell	0.158 (0.149–0.168)	0.527 (0.51–0.545)	0.182 (0.172–0.192)	0.278 (0.266–0.291)	1.077 (1.052–1.102)	0.016 (0.014–0.02)
2(a)1 Precursor Non-Hodgkin lymphoma, B-cell	0 (0–0.001)	0 (0–0.001)	0 (0–0.001)	0 (0–0.001)	0.004 (0.002–0.006)	0 (0–0.001)
2(a)2 Mature Non-Hodgkin lymphoma, B-cell	0.144 (0.135–0.154)	0.493 (0.477–0.511)	0.151 (0.142–0.16)	0.257 (0.246–0.27)	0.985 (0.961–1.009)	0.015 (0.013–0.019)
2(a)2.1 Chronic/Sm/Prolymphocytic/Mantle B-cell NHL	0.016 (0.013–0.02)	0.044 (0.039–0.049)	0.009 (0.007–0.011)	0.032 (0.028–0.037)	0.099 (0.092–0.107)	0.002 (0.001–0.003)
2(a)2.2 Lymphoplasmacytic lymphoma/Waldenstrom	0.002 (0.001–0.003)	0.006 (0.004–0.008)	0.001 (0–0.002)	0.003 (0.001–0.004)	0.018 (0.015–0.022)	0 (0–0.001)
2(a)2.3 Diffuse large B-cell lymphoma (DLBCL)	0.023 (0.019–0.027)	0.017 (0.014–0.021)	0.073 (0.067–0.08)	0.026 (0.022–0.03)	0.182 (0.172–0.193)	0.002 (0.001–0.003)
2(a)2.4 Burkitt lymphoma/leukemia	0 (0–0.001)	0 (0–0.001)	0 (0–0.001)	0.001 (0–0.002)	0.006 (0.004–0.008)	0 (0–0.001)
2(a)2.5 Marginal-zone lymphoma (MZL)	0.067 (0.061–0.073)	0.364 (0.35–0.379)	0.056 (0.05–0.062)	0.154 (0.144–0.163)	0.547 (0.53–0.565)	0.01 (0.008–0.012)
2(a)2.6 Follicular lymphoma	0.036 (0.032–0.041)	0.062 (0.056–0.068)	0.012 (0.01–0.015)	0.043 (0.038–0.048)	0.132 (0.123–0.141)	0.002 (0.001–0.003)
2(a)3 Non-Hodgkin lymphoma, B-cell, NOS	0.013 (0.011–0.016)	0.034 (0.029–0.038)	0.03 (0.026–0.035)	0.02 (0.017–0.024)	0.088 (0.081–0.095)	0.001 (0–0.002)
2(b) Non-Hodgkin lymphoma, T-cell	0.036 (0.032–0.041)	0.001 (0–0.002)	0.003 (0.002–0.005)	0.001 (0.001–0.003)	0.014 (0.011–0.017)	0 (0–0.001)
2(c) Non-Hodgkin lymphoma, unknown lineage	0.004 (0.003–0.006)	0.008 (0.006–0.01)	0.004 (0.002–0.005)	0.003 (0.002–0.004)	0.019 (0.016–0.023)	0 (0–0.001)
3 Composite Hodgkin lymphoma and NHL	0 (0–0.001)	0 (0–0.001)	0.001 (0–0.002)	0 (0–0.001)	0.001 (0–0.002)	0 (0–0.001)
4 Lymphoid neoplasm, NOS	0.013 (0.01–0.016)	0.03 (0.026–0.034)	0.041 (0.037–0.046)	0.013 (0.01–0.016)	0.07 (0.064–0.076)	0.001 (0–0.002)

Lymphoma occurred mainly unilaterally (90.7%, N = 16,216), although intraocular lymphoma was the most common site to present bilaterally (15%), contrary to the eyelid (2.7%). Most patients presented with mature Non-Hodgkin B-cell lymphoma (78.1%), representing the highest rate among all lymphoma subgroups with 2.2 ppm (2.16–2.24). Its subtypes, marginal zone lymphoma (MZL), diffuse large B-cell lymphoma (DLBCL), and follicular lymphoma, were the most common subtypes, with 45.7, 12.3, and 11.0%, respectively (Table 2). MZL characterized all extra-ocular sites, representing 61.5% of conjunctival tumors, 49.4% of lacrimal gland malignancies, and 44.6% of orbital lymphomas. MZL occurred second (22.6%) in intraocular lymphomas after DLBCL (Supplementary Table 1).

Follicular lymphoma affected the eyelid in 16.5% of cases, with a percentage similar to that of T-cell non-Hodgkin lymphoma. T-cell lymphoma was characteristically high in the skin against its behavior at all other sites.

The incidence of all lymphoma types increases with age. Follicular lymphoma peaked in the age group of 80–84 and then declined after age 85 years in a pattern similar to conjunctival lymphomas.

The states of Connecticut and Hawaii had the highest rates of incidence (4 and 3.8 ppm, respectively), while West Virginia and Arizona showed the lowest incidence rates (1.5 and 1.8 ppm, respectively) (Fig. 1).

### Race and regions

The incidence was highest in Asia–Pacific Islanders with an ASIR of 2.83 (N = 863) and lowest in American Indian/Alaska Natives with an ASIR of 1.47 (95% CI 1.1–1.9) (N = 73). The incidence in Blacks and Hispanics was lower than in the white race (1.97 and 2.39 vs. 2.64 ppm). In the NPCR data set, the incidence was higher in metropolitan 2.67 (95% CI 2.62–2.72) than in non-metropolitan regions with ASIR of 2.2 ppm (95% CI 2.1–2.3) (Data not shown).

### Incidence trends

**Figure 2:**
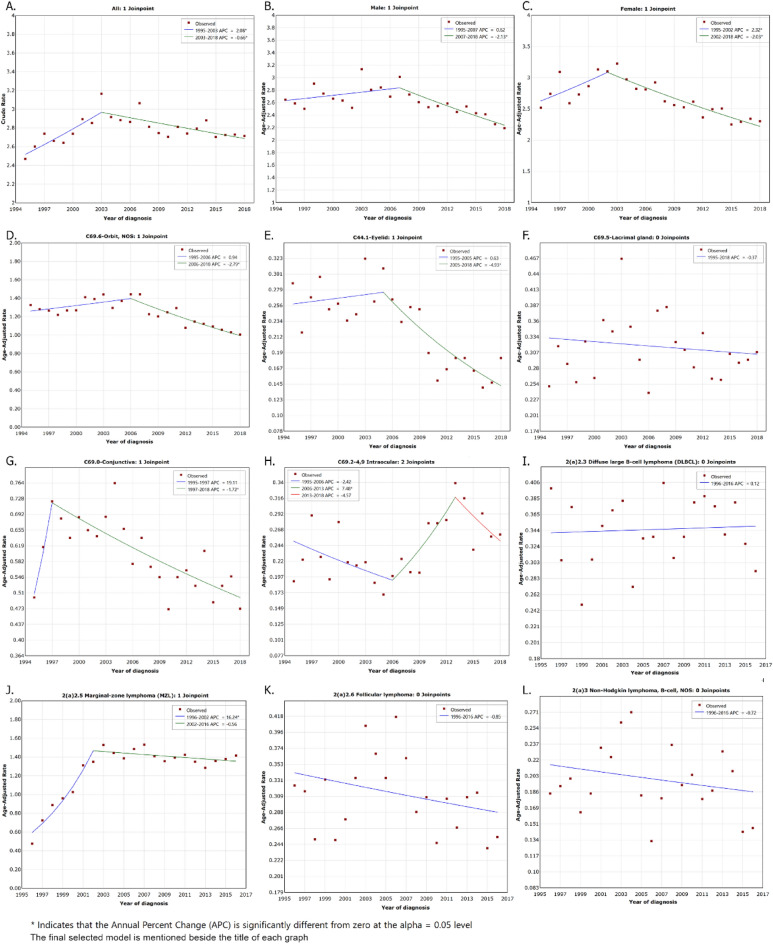
Trends in ocular lymphoma incidence rates between 1995 and 2018. A. Crude incidence rates in all patients B-L Age-standardized incidence rates according to sex (**B** and **C**), site (**D**–**H**), and histology (**I**–**L**). APC: Average percentage change. Please note the difference in y-axis.

The incidence rates in SEER databases 9, 13, and 18 fluctuated over the years and exhibited higher incidence rates than those in NAACCR (Supplementary Fig. 2). The NAACCR data showed a significant increase in both crude and age-standardized incidences between 1995 and 2003, with an average percent change (APC) of 1.58, followed by a stable significant decline from to 2003–2018, with an APC of − 1.71 (Fig. 2). The females showed an APC with an incidence of − 1.99, while men showed an APC of -1.45 since 2003. Further analysis showed a similar pattern of decline among males and females, all affected age groups, and geographical and topographical regions (Fig. 2, Supplementary Figs. 3 and 4). We investigated the presence of this pattern in all cases of NHL.

Interestingly, we found that the crude incidence rates of all-body NHL continued to increase until 2015, and then started to decline. However, the ASIR showed an increase until 2004, and then started to decline in two different stages: before and after 2015 (Supplementary Fig. 5). This trend showed a continuous decline in the roles of microscopic and histological diagnoses. We noticed a steady increase in the use of immunophenotyping since 2009 (Supplementary Fig. 6). Furthermore, there was an increase in the number of diagnosed extranodal marginal zone lymphomas during the study period.

### Treatment

Beam radiation is generally the most commonly used treatment modality, with a stable percentage (around 50%), but is less frequently used in intraocular lymphoma. Furthermore, the role of surgery has significantly increased over the years, although it is less frequent in intraocular diseases (Supplementary Fig. 7 and Supplementary Table 2). Biological response modifier therapy increased significantly, especially after 2011, accounting for 25% of its use in all cases. Chemotherapy and biological response modifiers are used more frequently in intraocular lymphomas, lacrimal glands, and orbital lymphomas. Enucleation was the final shelter in 7.64% (N = 128) of intraocular lymphomas. Excision plays a significant role in eyelid, conjunctival, lacrimal gland, and orbital lymphomas].

### Survival and survival trends

**Figure 3:**
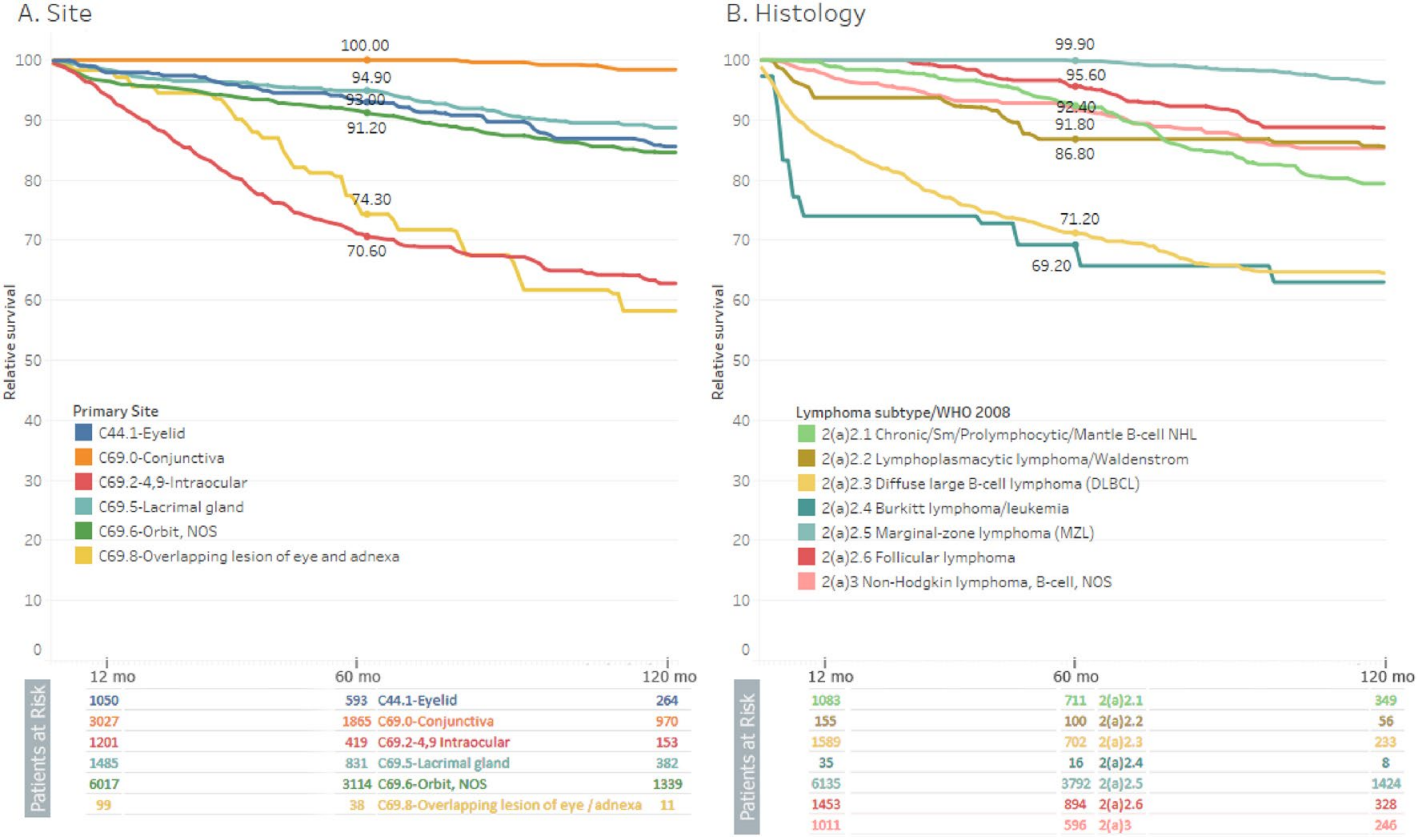
Cumulative relative survival of all patients between 1995 and 2018 according to site (A) and histology (B).

Survival rates were highest in conjunctival lymphoma (100%) and lowest in intraocular and overlapping lesions (70.60% and 74.30%, respectively). Regarding the types of lymphoma, marginal lymphoma had the most favorable survival (99.9%), while Burkitt lymphoma had the least favorable survival (69.20%) (Fig. 3). Patients who received (or) required radiation with surgery had the highest survival rate, followed by those who required radiation therapy only. Patients who required chemotherapy or chemotherapy combined with radiation treatment had the lowest overall survival (Supplementary Fig. 8).

**Figure 4:**
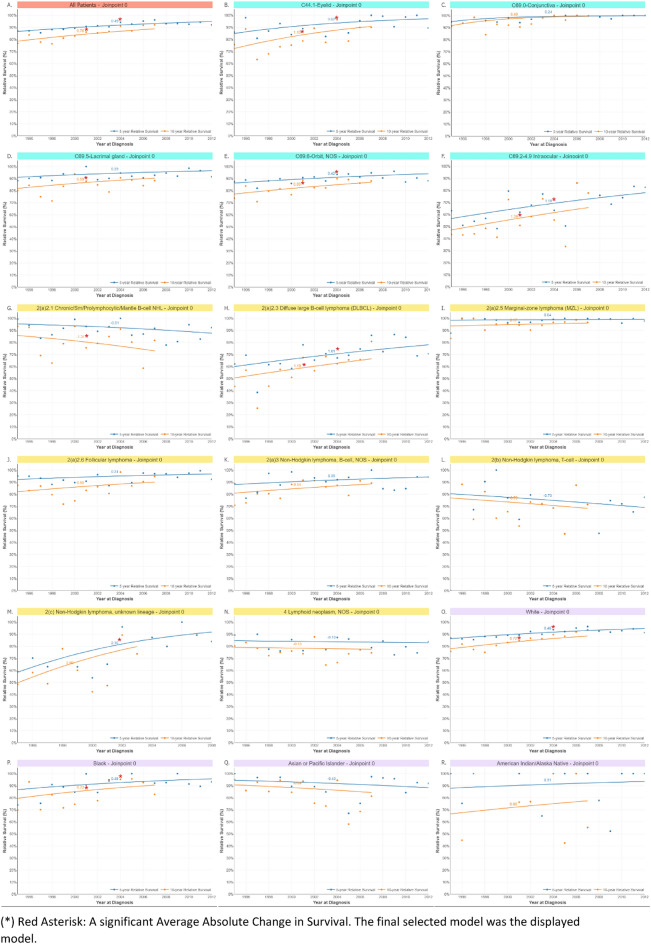
Trends of 5- and 10-year relative survival between 1995 and 2012; in all ocular lymphoma patients (**A**), according to site (**B**–**F**), histology (**G**–**N**), and race (**O**–**R**). *Red Asterisk: A significant Average Absolute Change in Survival. The final selected model was the displayed model.

The relative survival trend increased significantly with the year of diagnosis. We observed improved survival at all ocular topographical sites (Fig. 4). Regarding histological types, survival increased significantly in all lymphomas except for non-Hodgkin lymphoma T-cell, chronic, and Mantle B-cell NHL.

## Discussion

Previous studies conducted on the U.S. population and investigating ocular and orbital lymphoma used data extracted from The Surveillance, Epidemiology, and End Results (SEER) Program, which covers up to 30% of the U.S. population. The most recent study depended on the SEER database alone^11^. However, previous studies have shown possible variations between the SEER and NPCR databases or the combined database that covers 98–100% of the USA population^12–14^. The differences between SEER- and non-SEER-covered registries included discrepancies in urban–rural coverage, age composition, employment rate, and Medicare insurance coverage^14^. Therefore, these variations should be considered when the SEER results are generalized. USCS/NPCR and NAACCR databases have shown an advantage, especially in studying rare cancers or variants of malignancies. In our study, the results from the latter databases showed more stable incidence rates over the years, possibly due to the reduced effect of the reporting delay and less variation from inter-annual reports.

Initially, our study observed an increasing trend in the incidence of ocular lymphomas until 2003–2005, in line with prior reports, followed by a steady decline. This newly observed pattern could be attributable to either an inherent reduction in risk factors or a decrease in the proportion of solitary orbital lymphoma cases unassociated with other systemic lymphomas. The latter possibility may be elucidated by examining the total incidence of non-Hodgkin lymphoma, the growing trend of exploring systemic associations, and advancements in lymphoma diagnostic techniques, which could facilitate the classification of ocular disease as a component of systemic disease^15,16^. Conversely, the observed rise may result from the refinement of diagnostic tools or alterations in classification schemes that now encompass formerly benign adnexal NHLs, such as pseudolymphoma and reactive hyperplasia^9,17,18^. Our findings indicate a decline in both crude and age-adjusted incidence rates for ocular and orbital lymphomas, whereas only ASIR exhibits an early decline in all-body lymphoma. This implies that shifts in age distribution have impacted incidence rates, potentially due to the population pyramid’s waist transitioning toward an older demographic. However, this single factor may not adequately account for the observed variations and disparities between ocular/orbital and whole-body lymphomas, warranting further investigations. The incidence of ocular and orbital lymphomas has been the focus of a few previous studies. In Denmark, ophthalmic lymphoma increased from 0.86 ppm in 1981–1985 to 2.49 in 2001–2005, representing an annual average increase of 3.4%, which reached an incidence of 3.7 ppm between 2013 and 2017^19,20^. In Canada, the ASIR between 1992 and 2010 was 0.65 ppm per year, with an average annual increase in incidence rate of 4.5%^21^. Furthermore, age-standardized incidence rates in South Korea increased from 0.3 to 0.8 ppm between 1999 and 2016, with an annual per cent change of 6.61%^10^.

NHL is a common lymphoid neoplasm with broad heterogeneous subgroups driven by multiple mutations. It can originate from B or T lymphocytes inside or outside lymph nodes^22^. The worldwide age-standardized incidence of non-Hodgkin lymphoma in 2018 was 6.7 in males and 4.7 in females per 100,000^23^. However, due to the ongoing efforts of classification, not all statistical reports are aligned in describing lymphoma subtypes. In our study, the escalation was predominantly attributed to an increase in extranodal MZL, a trend that is not unique to the USA but appears prevalent throughout much of the Western world^8,9^. There has been debate on whether modifications in diagnostic techniques and updates in lymphoma classification could contribute to an apparent surge in lymphoma cases^8,24^. Notably, the classification of Extranodal MZL was officially established in 2001^24^.

Furthermore, the association of various lymphoma subtypes with distinct risk factors, such as viruses, including Epstein-Barr Virus and Human T-Lymphotropic Virus 1^25,26^, has been established. Notably, infections with Hepatitis C virus and Helicobacter pylori have been linked to an increased risk of developing non-Hodgkin lymphoma^27–29^, suggesting that the underlying mechanism may involve inflammation-induced tumorigenesis rather than direct viral effects. Additional risk factors have been elaborated upon in prior studies^28,30,31^. Although Chlamydia psittaci was previously identified as a significant risk factor, data from the Centers for Disease Control and Prevention (CDC) have indicated a decline in incidence rates in the United States since 1988^32^. Exposure to chlorinated hydrocarbons, notably trichloroethylene, benzines, and certain chemotherapeutic agents, has been implicated in lymphomagenesis^33–38^. Sjögren syndrome has been particularly associated with an increased risk of non-Hodgkin lymphoma, specifically Parotid MZL^39^. HIV infection has been associated with an increased risk of various lymphomas, including diffuse large B-cell lymphoma (DLBCL) and classical Hodgkin’s lymphoma^40^. Interestingly, the advent of highly active antiretroviral therapy (HAART) has not significantly altered the risk profile of non-Hodgkin lymphoma post-HAART era compared to the pre-HAART era^41^.

While Olsen et al. found that sex distribution correlates with specific lymphoma subtypes, their review detected that sex was only mentioned in 25% of published cases^3^. On the other hand, our study (based on NAACCR Data and including nearly all patients diagnosed between 1995 and 2018 n = 15,518 or 13,023)^1^, suggests a higher incidence in females in all locations and subtypes. Our study also confirms that, in the USA, Asia–Pacific islanders are more commonly affected by orbital lymphomas, which is consistent with previous findings by Moslehi et al.^42^.

Olsen et al. investigated lymphomas in the orbit but excluded the eyelid, conjunctiva, lacrimal sac, and eye globe^3^. Similar to our study, they concluded that orbital lymphoma is a common disease in the elderly. In addition, they showed that most tumors were of B cell origin (97%), of which extranodal MZL (59%) was the most common subtype, followed by DLBCL (23%). In our study, mature non-Hodgkin B-cell lymphoma had the highest rate among all lymphoma subgroups, with 2.2 ppm among its subtypes MZL in 45.7% of cases. We believe that earlier studies were affected by the introduction of the new classification systems at their time (Supplementary Fig. 6).

The age distribution varies among the different lymphoma subtypes; however, orbital lymphoma generally affects elderly patients (Tables 1 and 2). This applies especially to B-cell lymphomas, of which 73% are older than 50 years of age. Our statistics showed an evident increase in the number of patients affected by lymphomas (in all locations), starting from the age group of (40–59). However, most of these numbers doubled after the age of 60.

Advances in diagnostic techniques for primary ocular and orbital lymphomas, including immunohistochemistry, flow cytometry, cytokine analysis, and molecular diagnostics, have significantly enhanced our ability to identify and classify these malignancies accurately. Flow cytometry using immunophenotyping has been suggested to accurately detect different types of lymphoma^43^. Similarly, our study indicated a rising trend toward using immunophenotyping and genetic tests to confirm the diagnosis of ocular and adnexal lymphomas, which has increased significantly in recent years. However, the use of traditional biopsy techniques began to decrease in 2009.

To address the limitations of variability in diagnostic capabilities and pathologist expertise across institutions, especially in lymphoma diagnosis, and the missed opportunity for enhancing diagnostic reliability through standardized assessments, the incorporation of telepathology and AI tools in future investigations could be pivotal. Telepathology has been shown to enable the real-time interpretation of tissue biopsies and immunohistochemistry, facilitate accurate diagnoses, and potentially standardize assessments^44,45^. Additionally, AI tools have the potential to enhance diagnostic accuracy by leveraging large datasets and transfer learning techniques to improve classification performance significantly^46,47^.

In addition, the use of AI has already shown promise in supporting pathologists and in improving diagnostic efficiency, which could address the variability in diagnostic capabilities^48^.

Beam radiation is the most common treatment modality. Chemotherapy use rose significantly until 2011, when it declined (Supplementary Fig. 7). However, biological response modifier therapies such as monoclonal antibodies have been used extensively since 2012. While surgical interventions remained within their limits, patients treated with radiation followed by excision showed the highest survival rate throughout the years, followed by solo radiation therapy. R-CHOP remains the mainstay of treating diffuse large B-cell lymphoma; many studies have investigated the addition of biological response modifiers, yet relapse remains the main issue for many patients^49^.

In contrast, solo chemotherapy and chemotherapy combined with radiation showed the worst survival. Most likely, they die from systemic involvement, where ocular lymphomas are more often associated with CNS lymphoma, while extraocular lymphomas can be approached effectively with excision, irradiation, and chemotherapy. Our study indicates a significant improvement in the 5-year relative survival for all types of ocular and adnexal lymphomas, with better outcomes for recently diagnosed patients, which can be explained by recent advances in treatment modalities. This is consistent with the results of previous published studies^50,51^. Although survival has increased due to ongoing research focusing on early detection and the development of new treatment options holds promise for enhancing patient survival rates, the most effective approach to treating primary intraocular lymphoma remains under investigation, necessitating a multidisciplinary strategy involving both ophthalmologists and neuro-oncologists.

Nevertheless, lymphoma care has improved dramatically over the past 20 years. With new personalized treatment options such as CD20-targeting chemo-immune therapies, signal transduction inhibitors, CAR-T-cells, and hematopoietic stem cell transplantation, and in part also checkpoint inhibitors, the survival rate as well as the progression-free rate have improved dramatically^52–56^. Furthermore, protocols for radiation, systemic therapy, and a combination of both have been improved in previous studies^57,58^. The hope for cure from lymphoma, prolonged lifetime, and enhanced quality of life are features of the development of lymphoma care within this period, which must be taken into account in the analysis of these data.

Conjunctival lymphoma had the best cumulative survival rate, followed by eyelid and lacrimal glands. However, Intraocular tumors demonstrated the poorest survival rates, which is compatible with earlier studies that indicated the same results on a smaller scale^59^.

The difference in prognosis between intraocular lymphomas and orbital lymphomas may be attributed to several factors. One possible factor is the location and extent of disease. Intraocular lymphomas are confined to the eye, whereas orbital lymphomas can involve the orbital region surrounding the eye. Extraorbital disease in orbital lymphoma may indicate a more advanced stage and a higher risk of systemic spread, leading to poorer prognosis^60^.

Another factor that may contribute to the difference in prognosis is the histological subtype of the lymphoma. Intraocular lymphoma is often associated with a high-grade histological subtype such as diffuse large B-cell lymphoma, which is generally associated with a poorer prognosis^61^. In contrast, orbital lymphoma, particularly MALT lymphoma, is often associated with a low-grade histological subtype and generally has a better prognosis^3^.

Furthermore, treatment approaches for ocular and orbital lymphomas may also contribute to differences in prognosis. Intraocular lymphoma is typically treated with chemotherapy and radiation therapy^3^. However, the blood-retinal barrier can limit the effectiveness of systemic chemotherapy in treating intra-ocular lymphoma^62^. In contrast, orbital lymphoma is more accessible to surgical resection, which may improve local disease control^3^. Additionally, targeted therapies, such as rituximab, have shown promising results in the treatment of orbital lymphoma^63^.

Regarding the types of lymphoma, Burkitt lymphoma showed the worst overall survival (69.20%) between 1995 and 2018, followed by diffuse large B-cell lymphoma, which might be related to the aggressive pattern of these tumors and their relationship with other systemic manifestations^64^. However, marginal cell lymphoma has the most favorable outcomes.

Our study, like registry-based research, has some limitations. All participants were confirmed to have primary ocular or orbital lymphomas. Owing to data accessibility constraints, we were unable to investigate the occurrence of systemic lymphomas subsequent to the initial index lymphoma or synchronously associated lymphomas. The study’s capability to deduce the underlying causes for these trends is constrained by limited data on diagnostic and treatment patterns across the study period. The retrospective nature of our study and its reliance on multiple population databases may not fully capture the evolution of diagnostic methodologies or their impact on diagnosis and treatment. In addition, ICD-O-3 topography coding does not provide codes for all orbital or ocular regions’ sub-localizations, making it difficult to delinate the impact of the disease on minute ocular and orbital structures. Moreover, potential variability in reporting styles and treatment approaches across different data sources could introduce an element of heterogeneity that challenges the interpretation of trends over time. This limitation underscores the need for further research using more standardized data collection methods to better understand the etiopathogenesis of OAL and the impact of diagnostic and treatment strategies on patient outcomes. The registry may not be sensitive enough to gather all details of the management and follow-up of patients, including second-associated cancers^4^.

In conclusion, our study showed an increase in ocular and orbital lymphoma incidence until 2002, followed by a decrease in incidence. It also shows a discrepancy between the various states in terms of incidence. However, improved survival was observed in different ocular sites, and most lymphoma types were associated with the integration of new management approaches. Future research should aim to elucidate the potential link between various risk factors, such as infections, and observed epidemiological trends in ocular and orbital lymphomas.

## Methods

### Data sources and study population

The study utilized data collected from multiple databases: The North American Association of Central Cancer Registries (NAACCR), the United States Cancer Statistics Program (USCS), the National Program of Cancer Registries (NPCR), and the Surveillance Epidemiology and End-results Program of the National Cancer Institute (SEER) databases. For the NPCR incidence estimates, we only extracted data from registries that met the USCS standards between 2001 and 2018. For NAACCR Estimates, the study included the period from 01.01.1995 to 31.12.2018, covering the USA population. Patients’ inclusion and exclusion were conducted according to the diagram in Supplementary Fig. 1 (Numbers presented for NAACCR data).

The nationwide incidence of ocular and orbital lymphomas was estimated by independently analyzing the data from each source. The rates obtained from these sources were then compared to assess the consistency of the data across the different databases, and a comparison was highlighted in Supplementary Fig. 2. This demonstrated consistent incidence rates between NAACCR and NPCR, which were lower and more stable than the fluctuating rates observed in the SEER data. Age-standardized incidence rates among the states (Fig. 1) were determined using the NPCR database. The remaining analyses were performed using the NAACCR database.

ICD-O-3 codes were used to extract the data. Histological subtypes were determined using the WHO classification for lymphoma subtypes 2008^65^. We used the topography codes C44.1 “Eyelid” and C69.0-9 “Eye and its adnexa” to identify ocular sites. We grouped the ocular regions into eyelid, conjunctiva, intraocular, lacrimal gland, orbit-NOS, and overlapping lesions of the eye and adnexa.

The study adhered to the ethical guidelines of the Declaration of Helsinki and the International Conference on Harmonization. No ethical approval was required because of the open nature of the data that rendered the study non-human subject research, and they were anonymized before being delivered by the NAACCR and NPCR.

### Study variables

The following variables were extracted: age at diagnosis, sex, laterality, race, site, histology, state, region (either metropolitan or non-metropolitan), treatment (including surgery, chemotherapy, radiation, and biological response modifiers), method of confirmation of death, survival and survival time, and year of diagnosis.

### Incidence and survival rates

Incidence rates were calculated for 1,000,000 individuals. The age-standardized incidence rates were adjusted to U.S. 2000 standard population (single ages to 84—Census P25-1130)^66^. We calculated the age-standardized incidence rates (ASIR) and average percentage change (APC) to measure the annual change in incidence. The aim of presenting the SEER rates was to compare the previous results that used the 9 and 13 SEER cancer registries (which cover 10–30% of the USA population) to the currently available data, which covers up to 100% of the population (Supplementary Fig. 2).

We estimated relative survival using the actuarial method and Ederer II for cumulative expected estimates^67^. We used the U.S. rates file “U.S. by SES/geography/race (NHW, NHB, NHAIAN, NHAPI, HISP) 1992–2016, Ages 0–99, State-county (modelled by varied state-county-socioeconomic status)” to estimate the expected relative survival. We included only patients with malignant tumors and excluded patients with diagnoses through death certificates only and those who were alive with no survival time from the analysis. We excluded years that missed standard errors (e.g., 2018) when calculating trends when required.

### Software

We used SEER*Stat version 8.4.0.1 software to extract and analyze the data^68^. JoinPoint 4.9.1.0, was used to calculate the regression of the incidence trends over the study period and to fit the best model^69^. We used the JPSurv online software to calculate survival trends based on the year of diagnosis and chose the best-fitting model^70^. We used the Tableau program version 2021.2.0, to plot individual graphs^71^. A *p*-value of 0.05 was considered significant.

### Supplementary Information

Supplementary Information.

## Data Availability

The data supporting the findings of this study are available from [North American Association of Central Cancer Registries. (NAACCR) on www.naaccr.org] but restrictions apply to the availability of these data, which were used under license for the current study, and so are not publicly available. However, the data are available from the authors upon reasonable request and with permission from [North American Association of Central Cancer Registries. (NAACCR) on www.naaccr.org].

## Abbreviations

ASIR: Age-standardized incidence rate
APC: Average percent change
BMT: Bone Marrow therapy
DLBCL: Diffuse large B-cell lymphoma
HAART: Highly active antiretroviral therapy
HISP: Hispanics
MZL: Marginal zone B-cell lymphoma
NAACCR: North American Association of Central Cancer Registries
NHIA: NAACCR Hispanic Identification Algorithm
NHL: Non-Hodgkin lymphoma
NHW, NHB, NHAIAN, NHAPI: Non-Hispanic whites, blacks, Indian/Alaska native, Asian/Pacific Islander
NPCR: National Program of cancer registries
PPM: Persons per million
RS: Relative survival rates
SEER: Surveillance Epidemiology and End-results Program of National Cancer Institute
SES: State-county- socioeconomic status
USCS: United States cancer statistics program
CDC: Center of disease control
