# Supplementary material for: Declining incidence and improving survival of ocular and orbital lymphomas in the US between 1995 and 2018
Source: Sci Rep. 2024 Apr 3;14:7886. doi: 10.1038/s41598-024-58508-7 (PMC10991268; doi:10.1038/s41598-024-58508-7)
Supplement: Supplementary file 1 — Supplementary Information. [file 41598_2024_58508_MOESM1_ESM.docx]

# Supplementary

Supplementary Figure 1**:** Flow chart of the patients' cohort definition


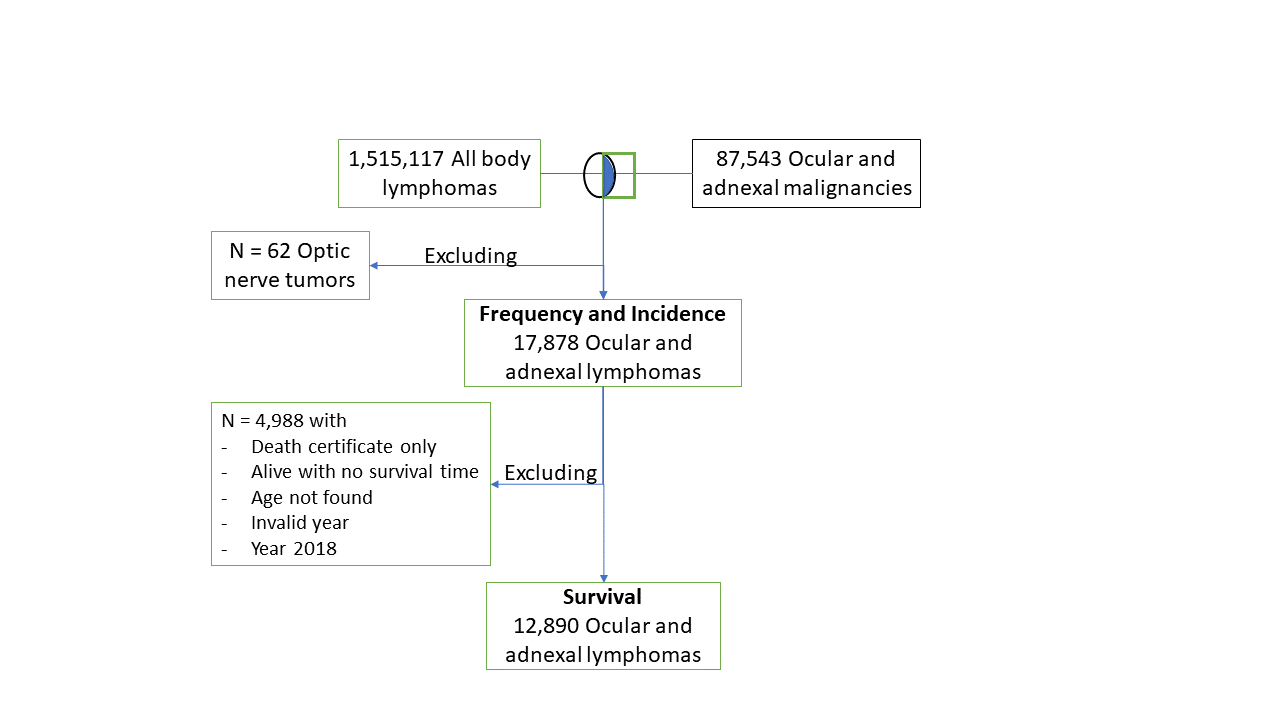


Supplementary Figure 2: Comparison of the trends in incidence rates over the years between different population-based databases.


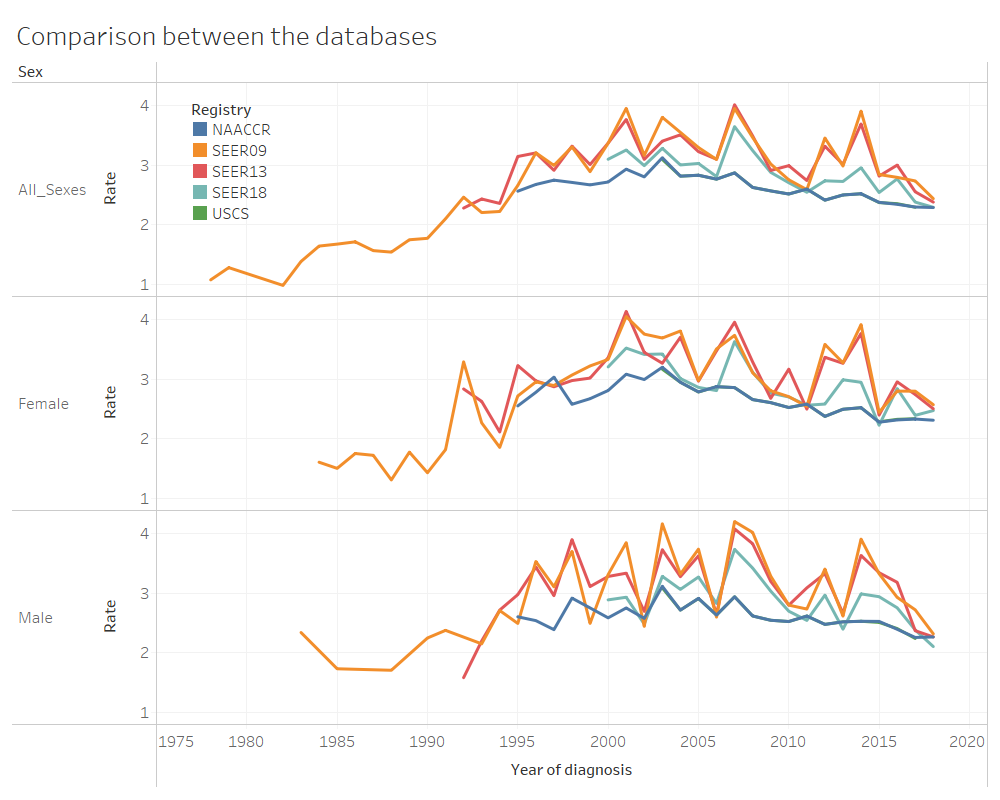


NAACCR: North American Association of Central Cancer Registries, SEER: Surveillance, Epidemiology, and End Results program. USCS: United States Cancer Statistics Division of Cancer Prevention and Control, National Program of Cancer Registries (NPCR), Centers for Disease Control and Prevention (CDC)

Note: NAACCR and USCS lines are overlapping, Rate is per million

Supplementary Figure 3. Trends in age-adjusted incidence of ocular lymphoma according to age group. A. 0-19 years, B. 20-39 years, C. 40-59 years, D. 60-79 years, E. 80+ years.


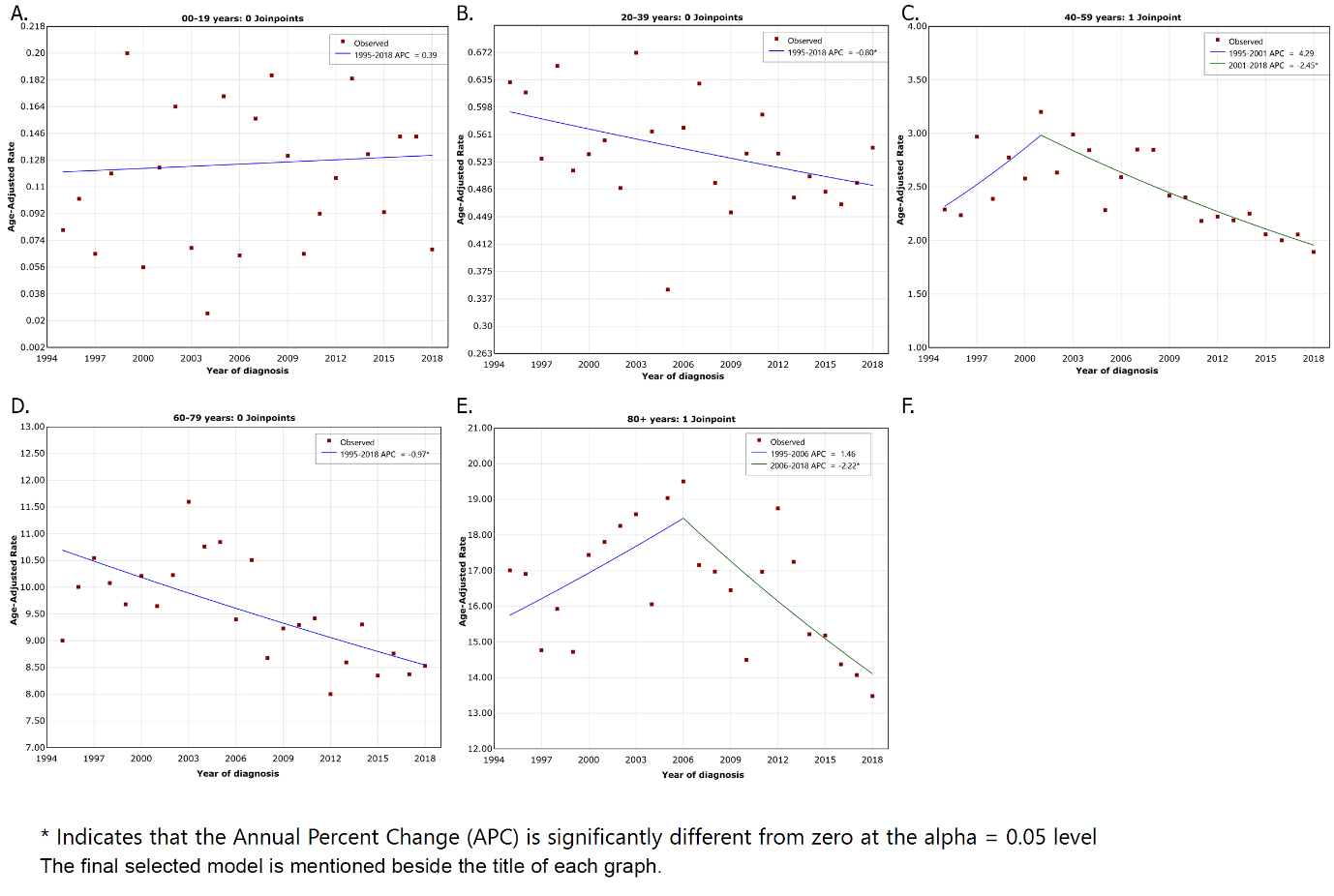


Supplementary Figure 4: Age-standardized rates according to Region, Race/Origin, and Site.


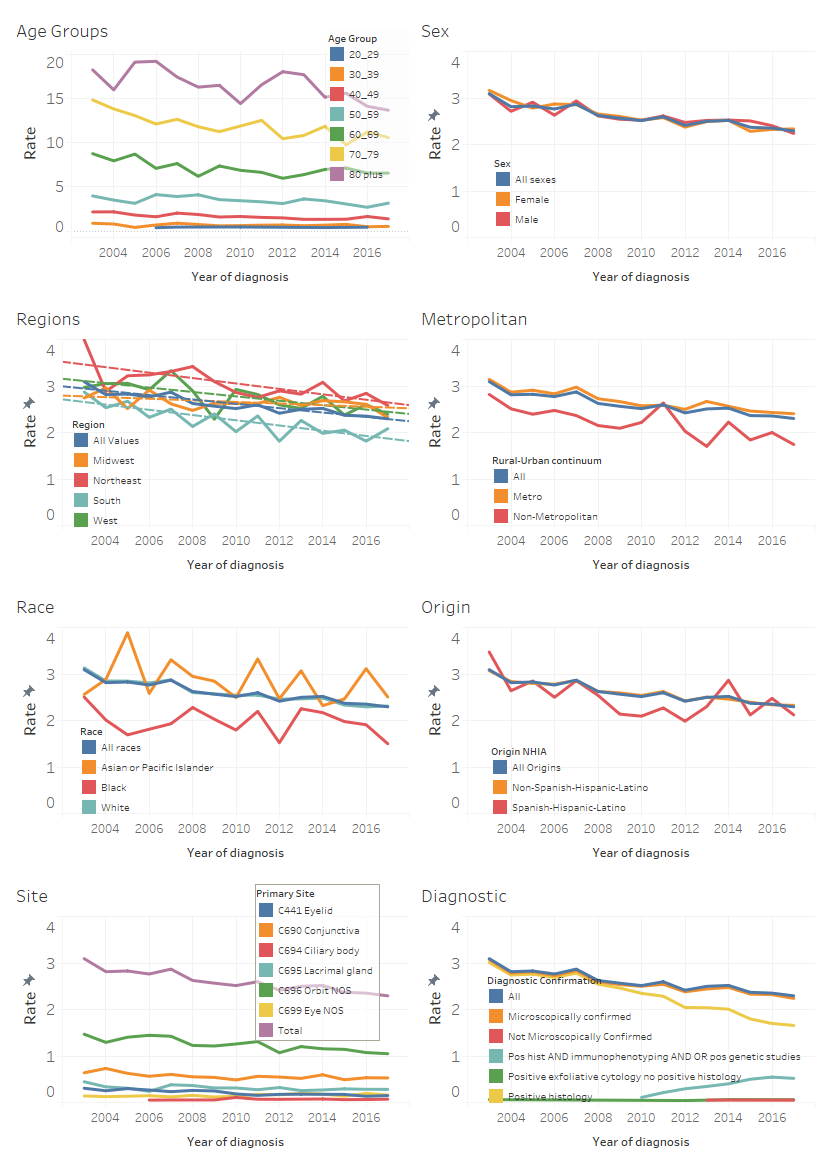


Supplementary Figure 5. Incidence trends of all non-Hodgkin lymphomas between 1995-2018. A. Crude rate, B. Age-Adjusted rate.

**A.**


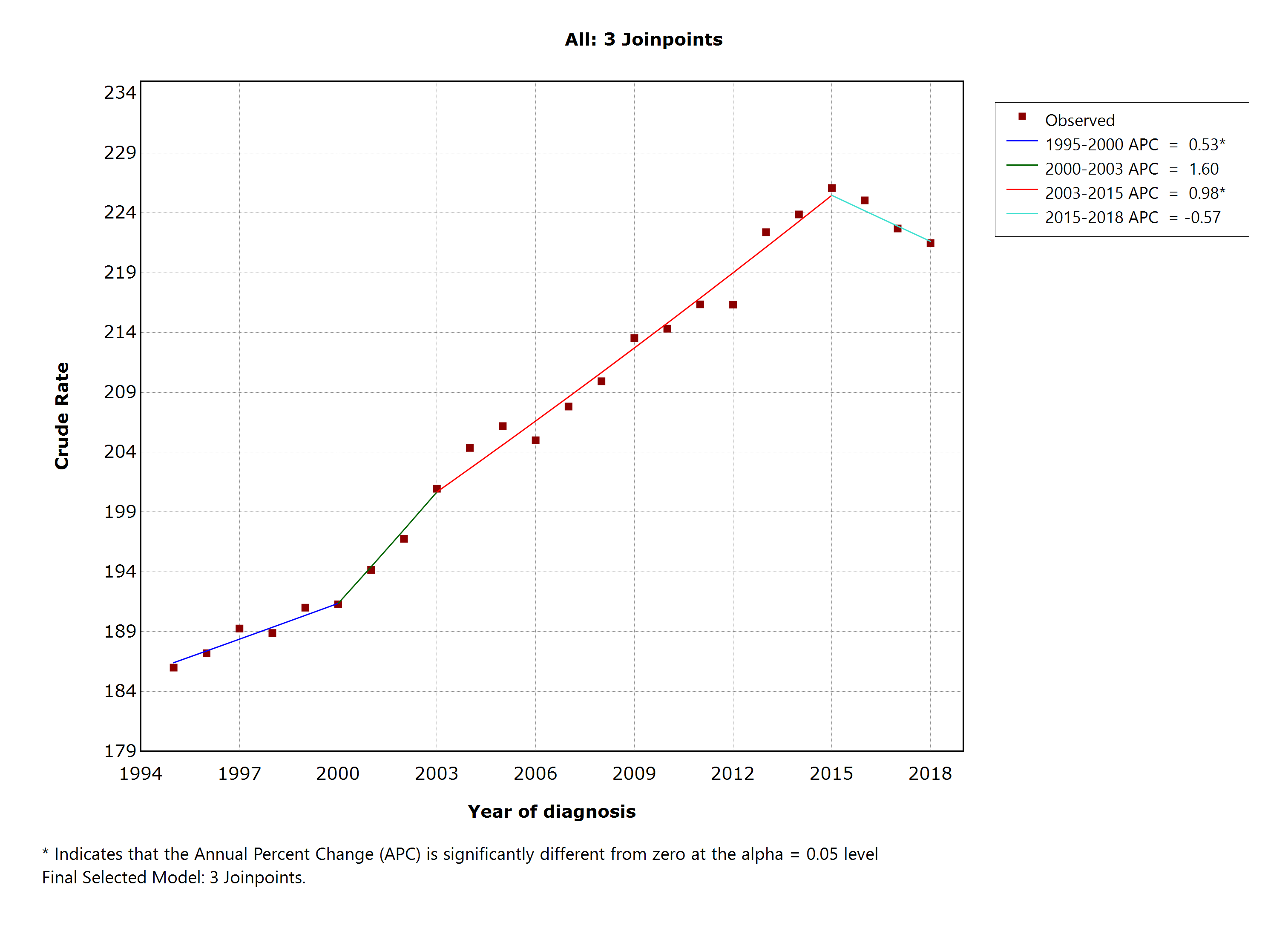


**B.**


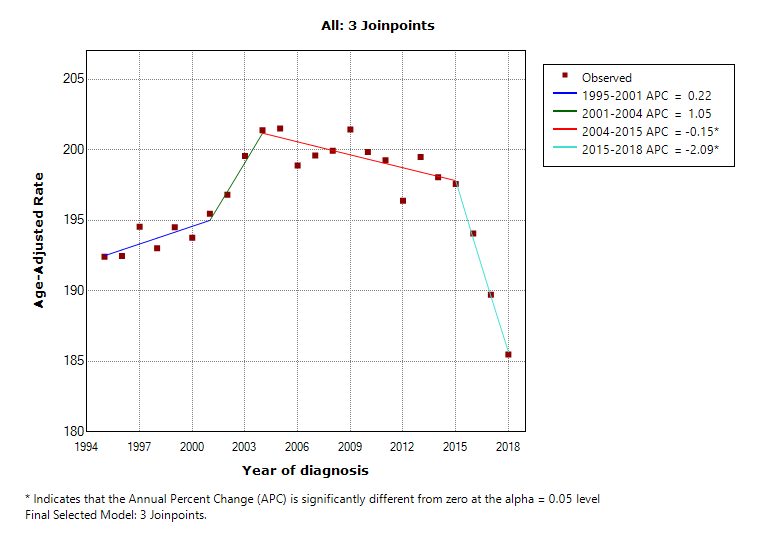


Supplementary Figure 6: The frequency of use of A. different main diagnostic confirmatory modalities with all ocular/orbital lymphoma patients B. Main histological subtypes between 1995-2017.


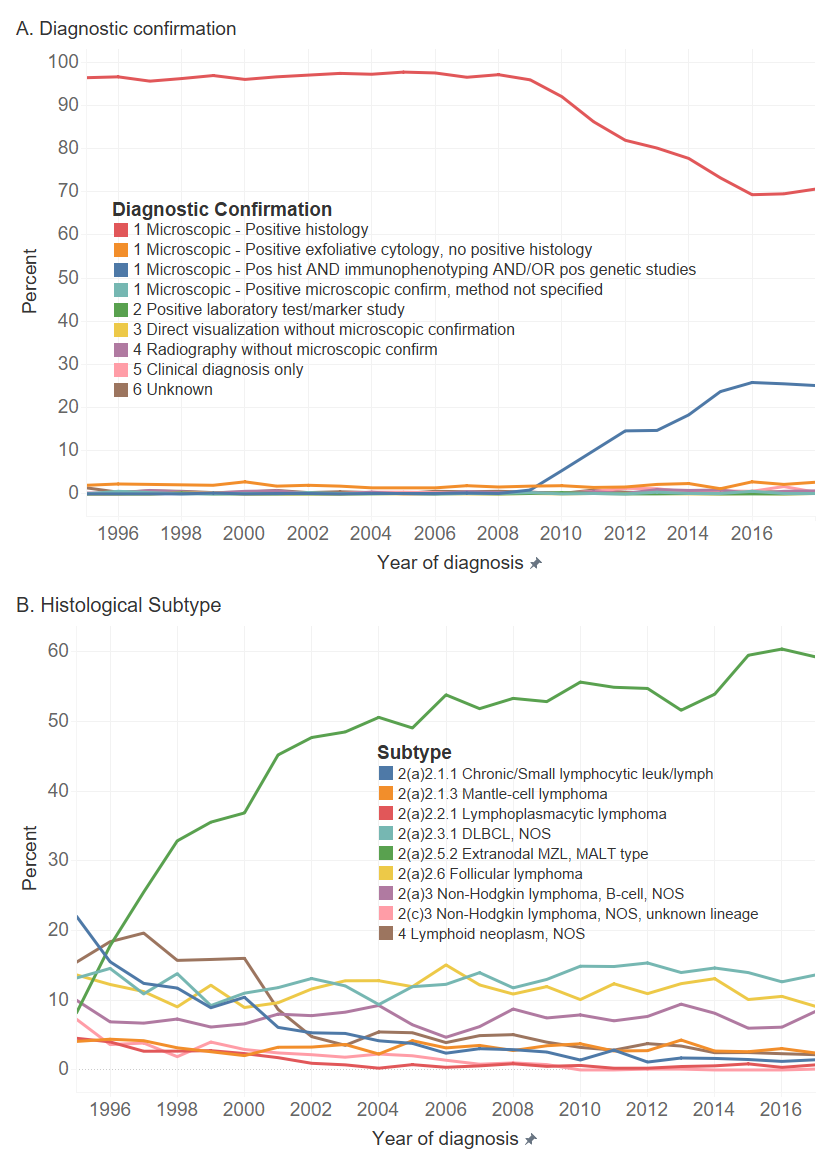


MZL: Marginal Zone Lymphoma, MALT: Mucosa associated lymphoid tissue, DLBCL: Diffuse large B cell lymphoma, NOS: Non-otherwise specified

Supplementary Figure 7: Frequency of use of different treatment modalities in all ocular lymphoma patients 1995-2017.


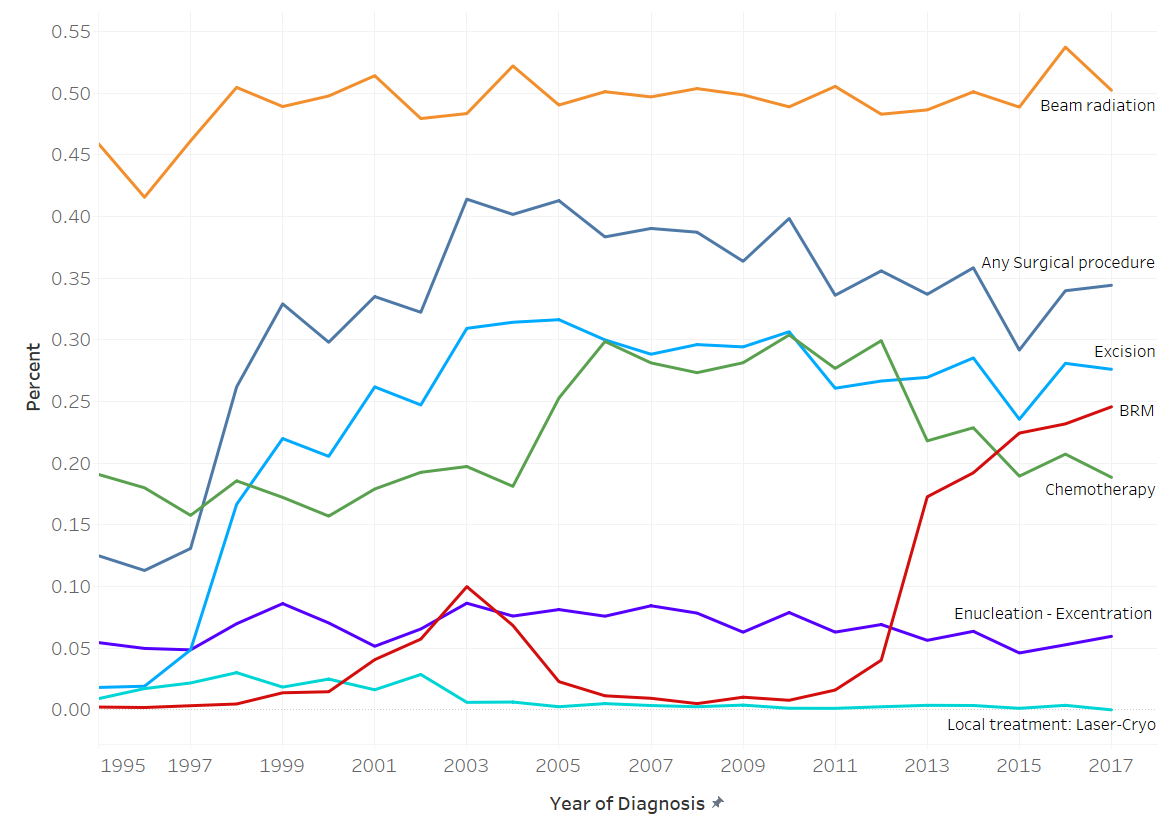


Supplementary Figure 8. Cumulative relative survival of all patients between 1995-2018 according to treatment.


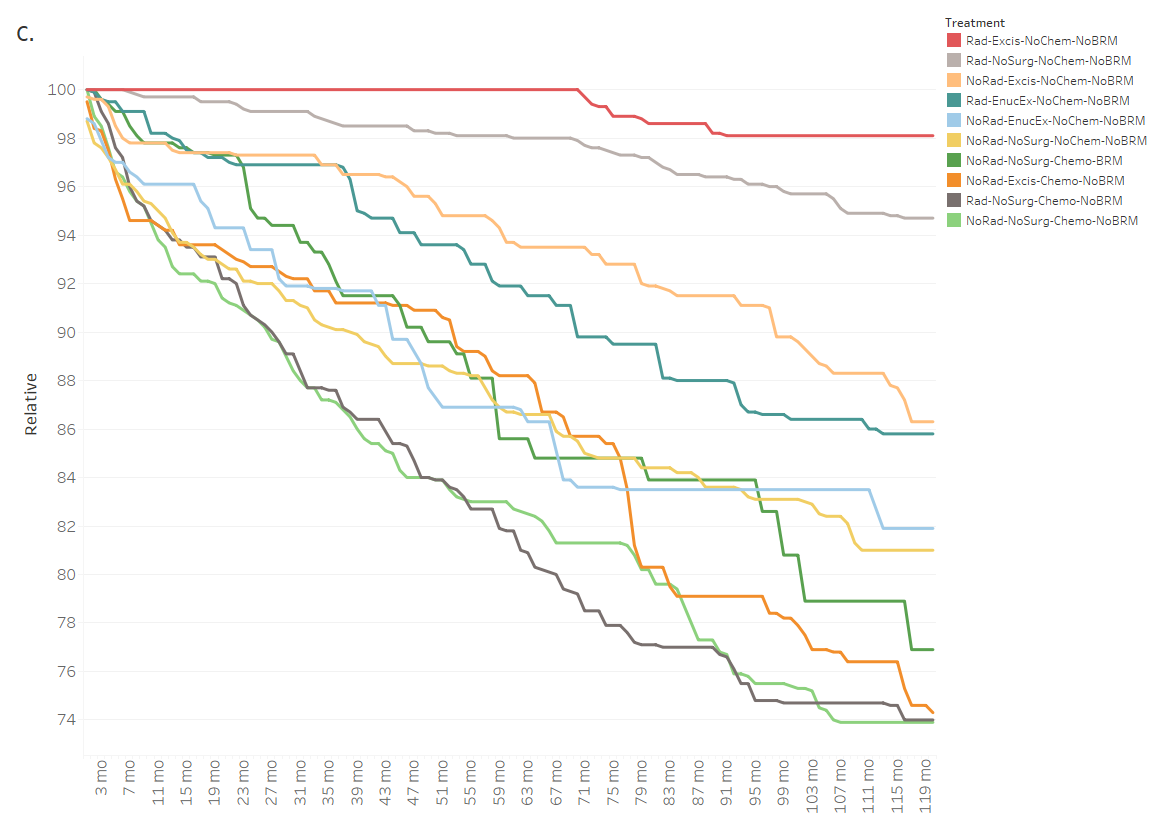


Acronyms: mo.: months; Rad.: Radiation; Excis.: Excision; Chem.: Chemotherapy; Surg.: Surgery; BRM: Biological Response Modifiers.

Supplementary Table 1: Patients’ Distribution according to histological subtype

| **Lymphoma subtype/WHO 2008** | **Eyelid** | **%** | **Conjunctiva** | **%** | **Intraocular** | **%** | **Lacrimal gland** | **%** | **Orbit, NOS** | **%** | **Overlapping lesions** | **%** | **Total** | **%** |
| --- | --- | --- | --- | --- | --- | --- | --- | --- | --- | --- | --- | --- | --- | --- |
| Lymphoid Neoplasm | 1439 | 95.5% | 3816 | 95.7% | 1581 | 94.4% | 2020 | 94.7% | 8077 | 95.6% | 123 | 93.9% | 17056 | 95.4% |
| 1 Hodgkin Lymphoma | 1 | 0.1% | 3 | 0.1% | 1 | 0.1% | 2 | 0.1% | 8 | 0.1% | 0 | 0.0% | 15 | 0.1% |
| 2 Non-Hodgkin lymphoma (NHL) | 1353 | 89.8% | 3614 | 90.7% | 1290 | 77.0% | 1929 | 90.4% | 7590 | 89.9% | 117 | 89.3% | 15893 | 88.9% |
| 2(a) NHL, B-cell | 1076 | 71.4% | 3555 | 89.2% | 1244 | 74.3% | 1903 | 89.2% | 7369 | 87.2% | 113 | 86.3% | 15260 | 85.4% |
| 2(a)1 **Precursor** NHL, B-cell | 2 | 0.1% | 1 | 0.0% | 3 | 0.2% | 2 | 0.1% | 23 | 0.3% | 0 | 0.0% | 31 | 0.2% |
| 2(a)2 **Mature** NHL, B-cell | 985 | 65.4% | 3327 | 83.5% | 1033 | 61.7% | 1765 | 82.7% | 6746 | 79.9% | 106 | 80.9% | 13962 | 78.1% |
| 2(a)2.1 Chronic/Sm/Prolymphocytic/Mantle B-cell NHL | 110 | 7.3% | 298 | 7.5% | 60 | 3.6% | 221 | **10.4%** | 682 | 8.1% | 12 | 9.2% | 1383 | 7.7% |
| 2(a)2.2 **Lymphoplasmacytic** lymphoma/Waldenstrom | 13 | 0.9% | 37 | 0.9% | 5 | 0.3% | 17 | 0.8% | 122 | 1.4% | 1 | 0.8% | 195 | 1.1% |
| 2(a)2.3 **Diffuse large B-cell** lymphoma (DLBCL) | 155 | 10.3% | 116 | 2.9% | 504 | **30.1%** | 176 | 8.3% | 1238 | **14.7%** | 13 | 9.9% | 2202 | **12.3%** |
| 2(a)2.4 **Burkitt** lymphoma/leukemia | 2 | 0.1% | 1 | 0.0% | 3 | 0.2% | 4 | 0.2% | 38 | 0.4% | 0 | 0.0% | 48 | 0.3% |
| 2(a)2.5 **Marginal-zone** lymphoma (MZL) | 457 | **30.3%** | 2451 | **61.5%** | 378 | **22.6%** | 1053 | **49.4%** | 3764 | **44.6%** | 66 | **50.4%** | 8169 | **45.7%** |
| 2(a)2.6 **Follicular** lymphoma | 248 | **16.5%** | 424 | **10.6%** | 83 | 5.0% | 294 | **13.8%** | 902 | **10.7%** | 14 | 10.7% | 1965 | 11.0% |
| 2(a)3 NHL, **B-cell, NOS** | 89 | 5.9% | 227 | 5.7% | 208 | **12.4%** | 136 | 6.4% | 600 | 7.1% | 7 | 5.3% | 1267 | 7.1% |
| 2(b) NHL, **T-cell** | 248 | **16.5%** | 8 | 0.2% | 21 | 1.3% | 9 | 0.4% | 91 | 1.1% | 1 | 0.8% | 378 | 2.1% |
| 2(c) NHL, unknown lineage | 29 | 1.9% | 51 | 1.3% | 25 | 1.5% | 17 | 0.8% | 130 | 1.5% | 3 | 2.3% | 255 | 1.4% |
| 3 **Composite** Hodgkin lymphoma and NHL | 1 | 0.1% | 3 | 0.1% | 5 | 0.3% | 3 | 0.1% | 7 | 0.1% | 0 | 0.0% | 19 | 0.1% |
| 4 Lymphoid neoplasm, **NOS** | 84 | 5.6% | 196 | 4.9% | 285 | 17.0% | 86 | 4.0% | 472 | 5.6% | 6 | 4.6% | 1129 | 6.3% |
| Not known / Invalid | 68 | 4.5% | 170 | 4.3% | 94 | 5.6% | 113 | 5.3% | 369 | 4.4% | 8 | 6.1% | 822 | 4.6% |

Supplementary Table 2. Treatment modalities among different sites

| **Treatment Modality** | C441 - Eyelid | | C690 - Conjunctiva | | C69.2,4,9 Intraocular | | C69.5 -Lacrimal gland | | C69.6 - Orbit NOS | | C69.8 - Overlapping lesions | |
| --- | --- | --- | --- | --- | --- | --- | --- | --- | --- | --- | --- | --- |
| **Surgery** | N | Col.% | N | % | N | % | N | % | N | % | N | % |
| No surgical procedure of primary site | 683 | 45.32 | 2398 | 59.86 | 1043 | 62.27 | 1176 | 55.13 | 4650 | 55.06 | 78 | 59.54 |
| Local treatment: Laser / Cryotherapy | 20 | 1.33 | 32 | 0.8 | 22 | 1.31 | 9 | 0.42 | 63 | 0.75 | 2 | 1.53 |
| Excision | 557 | 36.96 | 1020 | 25.46 | 262 | 15.64 | 558 | 26.16 | 2067 | 24.47 | 31 | 23.66 |
| Enucleation, Exenteration, Removing primary site | 49 | 3.25 | 91 | 2.27 | 128 | 7.64 | 165 | 7.74 | 738 | 8.74 | 8 | 6.11 |
| Unknown | 198 | 13.14 | 465 | 11.61 | 220 | 13.13 | 225 | 10.55 | 928 | 10.99 | 12 | 9.16 |
| **Radiation** |  |  |  |  |  |  |  |  |  |  |  |  |
| No Radiation | 629 | 41.74 | 1328 | 33.15 | 738 | 44.06 | 854 | 40.04 | 2888 | 34.19 | 49 | 37.4 |
| Radiation of any type | 647 | 42.93 | 2135 | 53.3 | 642 | 38.33 | 954 | 44.73 | 4335 | 51.33 | 66 | 50.38 |
| Unknown | 231 | 15.33 | 543 | 13.55 | 295 | 17.61 | 325 | 15.24 | 1223 | 14.48 | 16 | 12.21 |
| **Chemotherapy** |  |  |  |  |  |  |  |  |  |  |  |  |
| No Chemotherapy | 1102 | 73.13 | 3227 | 80.55 | 955 | 57.01 | 1466 | 68.73 | 5525 | 65.42 | 90 | 68.7 |
| Chemotherapy single or multiple | 238 | 15.79 | 437 | 10.91 | 572 | 34.15 | 492 | 23.07 | 2234 | 26.45 | 31 | 23.66 |
| Unknown | 167 | 11.08 | 342 | 8.54 | 148 | 8.84 | 175 | 8.2 | 687 | 8.13 | 10 | 7.63 |
| **Biological Response Modifier** |  |  |  |  |  |  |  |  |  |  |  |  |
| No | 1297 | 86.07 | 3545 | 88.49 | 1345 | 80.3 | 1802 | 84.48 | 7131 | 84.43 | 116 | 88.55 |
| Biological response modifier | 70 | 4.64 | 188 | 4.69 | 195 | 11.64 | 195 | 9.14 | 749 | 8.87 | 9 | 6.87 |
| Unknown | 140 | 9.29 | 273 | 6.81 | 135 | 8.06 | 136 | 6.38 | 566 | 6.7 | 6 | 4.58 |
| **Bone marrow transplantation** |  |  |  |  |  |  |  |  |  |  |  |  |
| None | 1330 | 88.25 | 3596 | 89.77 | 1484 | 88.6 | 1935 | 90.72 | 7655 | 90.63 | 121 | 92.37 |
| Bone marrow transplant | 0 | 0 | 4 | 0.1 | 5 | 0.3 | 2 | 0.09 | 11 | 0.13 | 0 | 0 |
| Not given | 3 | 0.2 | 3 | 0.07 | 3 | 0.18 | 1 | 0.05 | 4 | 0.05 | 0 | 0 |
| Stem cell harvest | 0 | 0 | 1 | 0.02 | 1 | 0.06 | 4 | 0.19 | 7 | 0.08 | 0 | 0 |
| Unknown | 174 | 11.55 | 402 | 10.03 | 182 | 10.87 | 191 | 8.95 | 769 | 9.1 | 10 | 7.63 |
| **Total** | 1507 | 100 | 4006 | 100 | 1675 | 100 | 2133 | 100 | 8446 | 100 | 131 | 100 |
